# Molecular Study of Quinolone Resistance Determining Regions of *gyrA* Gene and *parC* Gene*s in* Clinical Isolates of *Acintobacter baumannii Resistant* to Fluoroquinolone

**DOI:** 10.2174/1874285801812010116

**Published:** 2018-04-30

**Authors:** Maysaa El Sayed Zaki, Nermen Abou ElKheir, Mohamed Mofreh

**Affiliations:** Clinical Pathology Department, Mansoura Faculty of Medicine, Mansoura University, Mansoura, Egypt

**Keywords:** *A. baumannii*, Quinolone resistance, RFLP-PCR, Molecular mechanisms, Clinical isolates, Mutations

## Abstract

**Introduction:**

*Acinetobacterb aumannii* (*A. baumannii*) is an important pathogen in health care associated infections. Quinolone resistance has emerged in this pathogen.

**Aims & Objectives:**

The aim of the present study was to determine the presence of mutations of *gyrA* gene and *parC* genes by Restriction Fragment Length Polymorphism Polymerase Chain Reaction (RFLP-PCR) among clinical isolates of *A. baumanii*.

**Materials and Methods:**

The study was carried out on 140 clinical isolates of *A. baumannii*. The isolates were subjected to molecular study of mutations of *gyrA* gene and *parC* genes by RFLP–PCR beside determination of Minimal Inhibitory Concentration (MIC) by macro dilution tube method.

**Results:**

The isolates of *A. baumannii* were resistant to ciprofloxacine and levofloxacin at MIC >4 µg/ml. The most isolates had MIC >128 µg/ml (42.3%). All resistant strains to ciprofloxacin of *A. baumannii* had mutations in *gyrA* and *parC*. The most frequent mutations were combined mutations in both genes (85.5%) and 5% had single mutation either in *gyrA* or *parC*. The most frequently combined mutations were associated with MIC >128 µg/ml (42.3%).

**Conclusion:**

From this study we can conclude that resistance to ciprofloxacin was common in clinical isolates of *A. baumannii*. The most frequent mutations were present in *gyrA* and *parC*. However, mutations in *parC* alone were not uncommon. Further large scale studies are required to elucidate the resistance pattern of *A. baumannii* and its molecular mechanisms.

## 1 INTRODUCTION

*Acinetobacter baumannii (A. baumannii)* has emerged in the last years as an important pathogen causing health care associated infections. It is one of the so called “ESKAPE” bacteria known to have high antibiotics resistance (*Enterococcus faecium*, *Staphylococcus aureus*, *Klebsiella pneumoniae*, *Acinetobacter baumannii*, *Pseudomonas aeruginosa*, and Enterobacter species) [1]. *A. baumannii* is responsible for varieties of serious infections especially among patients in intensive care units such as blood stream infections, ventilator associated pneumonia, wound infections and urinary tract infections [2, 3].

*A. baumannii* has been associated with several antibiotics resistance not limited to Extended-Spectrum β-Lactamase. There are reports concerning the development of resistance to fluoroquinolones that represents a therapeutic target for this organism [4].

Fluoroquinolones act mainly on bacterial DNA topoisomerases enzymes class II (DNA gyrase) and class I. These enzymes are important for DNA replication in bacterial species. Fluoroquinolones inhibit the action of supercoiling in the bacterial cells mediated by both enzymes and result in impaired DNA replication therefore inhibiting cell division [5, 6].

The main mechanism of resistance to fluoroquinolones is the mutations of the genes that encode the subunits of DNA gyrase (*gyrA* and *gyrB*) and topoisomerase IV (*parC* and *parE*). The mutations involve mainly the Quinolone Resistance-Determining Regions (QRDRs) of *gyrA* gene and its homologous region of the *parC* gene. On the other hand, the mutations in *gyrB* and *parE* genes regions are of minor significance [7-9].

There are few data from Egypt on fluoroquinolone resistance among isolates of *A. baumanii* that involves the mutations in the genes of *gyrA* and *parC* genes. Therefore, the aim of the present study was to determine the presence of mutations in *gyrA* and *parC* by Restriction Fragment Length Polymorphism Polymerase Chain Reaction (RFLP-PCR) among clinical isolates of *A. baumanii*.

## 2 MATERIALS AND METHODS

The study was cross sectional prospective study that was performed in Mansoura University hospitals, Egypt from January 2016 till January 2017. The study included clinical samples from patients admitted to intensive care units (ICUs) with health care associated infections according to the CDC definitions. The study was approved by Mansoura faculty of Medicine ethical committee. Bacterial isolates were recovered from clinical samples according to the standard microbiological culture techniques. Biochemical identification of the *A. baumanii* isolates was based on Microscan automated system.

Antibiotics susceptibility tests for the isolates was carried out by agar disc diffusion method.

### 2.1 Antibiotics Discs Susceptibility

Antibiotics susceptibility was performed by disc diffusion method according to the Clinical and Laboratory Standards Institute guidelines [CLSI 2017]. The discs used for antibiotics susceptibility were ampicillin (2µg), ampicillin/sulbactam (10µg+10µg) cefepime (30µg), cefoxitin (30µg), ceftazidime (10µg), ciprofloxacin (1µg), gentamicin (30µg), imipenem (10µg), ceftriaxone (30 µg), meropenem (10 µg), levofloxacin (5μg) and trimethoprim/sulfamethoxazole (1.25/23.75μg), cefotaxime (30µg) (Oxoid).

### 2.2 Minimal Inhibitory Concentration (MIC) for Ciprofloxacin

Broth macrodilution method was used for the determination of MIC for ciprofloxacin among isolated *A. baumanii* according to the CLSI instructions. Serial dilutions of ciprofloxacin were prepared using Muller Hinton broth and bacterial isolates with concentration equal to 0.5 McFarland were added to each tube and incubated at 37^O^C for 18 hours. MIC was defined as the lowest concentration with no visible growth. *A. baumannii* isolates were considered as intermediate-resistant and full-resistant to ciprofloxacin when the MIC was 2 µg/ml and ≥4 µg/ml, respectively [10].

RFLP -PCR for Detection of Mutations in Gene’s *gyrA* and *parC*

### 2.3 DNA Extraction

Identified isolates of *A. baumannii* were subjected to DNA extraction by boiling method. Briefly colonies were dissolved in 1ml sterile distilled water and boiled for 10 minutes then centrifuged for another 10 minutes. The supernatant was preserved in sterile eppendorf at -20^O^C until amplification.

### 2.4 Amplification Procedures

Amplification procedure was performed by the use of 2 μl of the extracted DNA added to total mixture of 20 μl of amplification mixture supplied by Qiagen. The used primers were summarized in Table **1**. The programmed cycles for amplifications were described previously by Villa *et al*., 1995 and Villa *et al*., 1999 [7, 8].

Restriction Fragment length Polymorphism for Detection of Mutations in *gyrA* and *parC*.

*HinfI* (Promega) enzyme was used for the digestion of *gyrA* and *parC* in the amplified products of *A. baumannii*. The amplified products were purified by the use of QIA quick PCR purification kit (QIAGEN, Valencia, CA) according to the manufacturer’s protocol. The eluant was incubated at 37°C for 2.5 hours with 10 U of *HinfI*. The digests were then separated by electrophoresis in 1.5% agarose gel stained with ethidium bromide and photographed with UV illumination. The presence of 291 bp indicated the presence of mutation in *gyrA* and the presence of 52 bp indicated the presence of mutation in *parC*.

## 3 RESULTS

Total 140 isolates of *A. baumannii* were recovered from clinical isolates from ICUs during one year duration of the study. The majority of the isolates were recovered from wounds culture 60 (42.9%) followed by blood cultures 50 (35.7%) and urine cultures 30 (21.4%), (Table **2**).

There was a high resistance of the isolated *A. baumannii* to antibiotics with resistance to ampicillin (100%), ampicillin/sulbactam (100%), imipenem (100%), ceftazidime (99.3%), cefipime (96.4%). Resistance to ciprofloxacin was 92.3%., (Table **3**).

The isolates of *A. baumannii* with resistance to ciprofloxacine and levofloxacin had MIC >4 µg/ml. The most isolates had MIC >128 µg/ml (42.3%), (Table **4**).

All resistant strains of *A. baumannii* to ciprofloxacin had mutations in *gyrA* and *parC*. The most frequent mutations were combined mutations in both genes (85.5%) and 5% had single mutation either in *gyrA* or *parC*, (Table **5**).

The most frequent combined mutations was associated with MIC >128 µg/ml (42.3%), (Table **6**).

## 4 DISCUSSION

*A.* *baumannii* has emerged in last few years as an important pathogen of the health care associated infections. The principle pathogenicity of *A.* *baumannii* arises from its wide antibiotic resistance pattern making few options for its therapeutic management among which are flouroquinolone compounds [11].

In the present study *A. baumannii* was identified by the use of Microscan automated system. Generally, the automated system is an acceptable method for identification of this species compared with other automated and molecular systems such as Vitek 2 and Matrix-Assisted Laser Desorption Ionization-Time of Flight Mass Spectrometry (MALDI-TOF MS)-based VITEK MS [12].

For antibiotics susceptibility tests antibiotics discs diffusion method was used for screening of the resistance pattern of the isolated *A. baumannii* species.

All *A. baumannii* isolates were multidrug resistance according to the definition of Magiorakos & Perl 2008 [11]. Previous report supported the use of quinolone, ceftazidime and impinem as an empirical antibiotics therapy [13]. However, in the present study there was high resistance of the isolated *A. baumannii* to antibiotics with resistance to ampicillin (100%), ampicillin/sulbactam (100%), imipenem (100%), ceftazidime (99.3%), cefipime (96.4%). This pattern of antibiotics resistance is similar to that reported previously from Egypt on limited number of *A. baumannii* strains [14]. Therefore, this pattern of resistance limits the available empirical antibiotics therapy used for treating the infections caused by this pathogen. The factors associated with this resistance may be attributed to the overuse of these antibiotics as an empirical therapy with increase of antibiotics resistance through transmission of resistant genes [15]. The remaining therapeutic options are colistin (a relatively toxic drug), tigecycline (a bacteriostatic agent) that has a disadvantage when using in immunocompromised patients [16].

By disc diffusion and MIC the resistance to ciprofloxacin was 92.3%. This is similar to previous study reported in 2017 and more than another study in Egypt, (75%) [17].The resistance of isolated *A. baumannii* to ciprofloxacine has shown marked increase in the last few years in the developing countries with range from 95% up to 97.7% [3, 18, 19].

In the present study all isolates of A. resistant by disc diffusion method were resistant by MIC method. Similarly, Stone *et al*., 2007 [20] reported that disc diffusion method was a good rapid screening method to MIC for determination of antibiotics susceptibility.

Resistance of *A. baumannii* is mediated mainly by mutation in *gyrA* and *parC* genes [4, 15, 21, 22].

In the preset study, all resistant *A. baumanii* isolates to ciprofloxacin had mutations of *gyrA* gene and/or *parC* by PCR-RFLP analysis described by previous studies [7, 8].

This method detects the presence of *gyrA* and *parC* mutations at codon 83 and codon 80 with substitution of serine with leucine in *gyrA* and serine with leucine in *parC*.

The isolates of *A. baumanii* with MIC to ciprofloxacin more than 128 μg/mL had combined mutations in the *gyrA* and *parC*. These findings indicate that combined mutations are associated with high MIC resistance to ciprofloxacin. The combined mutations in these genes are associated with marked decrease in the binding affinity for quinolones in the resistant species [23-27].

The results of our study and the previous study from Egypt [4] suggested that these mutations are common in ciprofloxacin-resistant clinical isolates of *A. baumannii* and similar findings were reported in previous studies [7, 8, 12, 22, 28]. The study of mechanisms implicated in resistance of *A. baumannii* to ciprofloxacin should be clarified in different local epidemiological situations.

Though combined mutations of *parC* and *gyrA* are reported to be associated with a high-level of resistance to quinolones [8], four clinical isolates in our study had mutations in *parC* without mutations in *gyrA*, suggesting that *parC* may had a primary role in developing resistance to ciprofloxacin without mutation in *gyrA*. Similar findings were reported by Ardebili *et al*., 2015 [29].

All the isolates of the *A. baumanii* resistant to ciprofloxacine were associated with mutations in *gyrA* and *parC*. However, other mechanisms can be associated with this resistance such as changes in the efflux systems [30]. Further studies with large number of isolates are required to clarify the mechanisms associated with resistance of *A. baumanii*.

## CONCLUSION

 From this study we can conclude that resistance to ciprofloxacin is common in clinical isolates of *A.* baumannii. The most frequent mutations were those present in *gyrA* and *parC* genes. However, mutation in *parC* alone was not uncommon. Further, large scale studies are required to elucidate the resistance pattern of *A. baumannii* and its molecular mechanisms.

## Figures and Tables

**Table 1:** Primers sequences of the target genes.

Target Gene	Primer sequence (5'-3')	Amplicon size
*gyrA*	Forward AATCTGCCCGTGTCGTTGGT	343 bp
	Reverse GCCATACCTACGGCGATACC	
*parC*	Forward AAACCTGTTCAGCGCCGCATT	327 bp
	Reverse AAAGTTGTCTTGCCATTCACT	

**Table 2:** Distribution of *A. baumannii* isolates from clinical samples.

Sample	No.	%
Blood Culture	60	42.9
Wounds Culture	50	37.5
Urine Culture	30	21.4
Total	140	100%

**Table 3:** Antibiotics Resistance among isolates of A. *baumannii*.

Antibiotics	No.	%
Ampicillin	140	100
Ampicillin/sulbactam	140	100
cefepime	135	96.4
cefoxitin	134	95.7
ceftazidime	139	99.3
ciprofloxacin	130	92.9
gentamicin	100	71.4
imipenem	140	100
ceftriaxone	130	92.9
meropenem	140	100
levofloxacin	130	92.9
trimethoprim/sulfamethoxazole	124	88.6
cefotaxime	128	91.4

**Table 4:** A. *baumannii* resistance by MIC.

	**MIC** **µg/ml**						
**4** **No. %**	**8** **No. %**	**16** **No. %**	**32** **No. %**	**64** **No. %**	**128** **No. %**	**>128** **No. %**
**gyrA**	**35 26.9%**	**30 23.1%**	**2 1.5%**	**3 2.3%**	**2 1.5%**	**3 2.3%**	**55 42.3%**

**Table 5:** Genetic mutations among *A. baumannii* isolates.

Characteristics	N0.	%
*gyrA*	5	3.6%
parC	5	3.6%
Combined *gyrA* and *parC*	120	85.7%
Total	140	100%

**Table 6:** Distribution of gyrA and parC mutations among resistant *A. baumannii* in relation to MIC.

Characteristics	**MIC** **µg/ml**						
**4** **No. %**	**8** **N0. %**	**16** **N0. %**	**32** **No. %**	**64** **No. %**	**128** **N0. %**	**>128** **N0. %**
***gyrA***	**0 0%**	**0 0%**	**0 0%**	**3 2.3%**	**2 1.5%**	**0 0%**	**0 0%**
***parC***	**0 0%**	**0 0%**	**2 1.5%**	**0 0%**	**0 0%**	**3 23%**	**0 0%**
**Combined *gyrA* and *parC***	**35 26.9%**	**30 23.1%**	**0 0%**	**0 0%**	**0 0%**	**0 0%**	**55 42.3%**
